# LocaRDS: A Localization Reference Data Set

**DOI:** 10.3390/s21165516

**Published:** 2021-08-17

**Authors:** Matthias Schäfer, Martin Strohmeier, Mauro Leonardi, Vincent Lenders

**Affiliations:** 1 Department of Computer Science, University of Kaiserslautern, 67663 Kaiserslautern, Germany; 2 OpenSky Network, 3400 Burgdorf, Switzerland; lenders@opensky-network.org; 3 Armasuisse Science + Technology, 3703 Thun, Switzerland; 4 Department of Computer Science, University of Oxford, Oxford OX1 3QD, UK; 5 Department of Electronic Engineering, University of Rome Tor Vergata, 00133 Rome, Italy; mauro.leonardi@uniroma2.it

**Keywords:** localization, *positioning*, *self-positioning*, data set, TDoA, aircraft localization

## Abstract

The use of wireless signals for the purposes of localization enables a host of applications relating to the determination and verification of the positions of network participants ranging from radar to satellite navigation. Consequently, this has been a longstanding interest of theoretical and practical research in mobile networks and many solutions have been proposed in the scientific literature. However, it is hard to assess the performance of these in the real world and, more importantly, to compare their advantages and disadvantages in a controlled scientific manner. With this work, we attempt to improve the current state of art methodology in localization research and to place it on a solid scientific grounding for future investigations. Concretely, we developed LocaRDS, an open reference data set of real-world crowdsourced flight data featuring more than 222 million measurements from over 50 million transmissions recorded by 323 sensors. We demonstrate how we can verify the quality of LocaRDS measurements so that it can be used to test, analyze and directly compare different localization methods. Finally, we provide an example implementation for the aircraft localization problem and a discussion of possible metrics for use with LocaRDS.

## 1. Introduction

In mobile wireless networks, three related problems have received considerable attention in the scientific literature and industry applications: positioning (or localization), self-positioning and location verification. In *positioning*, the wireless network aims to determine the location of a remote and typically mobile node, whilst in *self-positioning*, the remote node seeks to find out its own (absolute or relative) position. A common pattern in systems where self-positioning is used to establish location awareness among nodes is that location information is exchanged across the network. In scenarios where location is used in safety-critical services or security-critical services, the need for *location verification* arises. Here, a set of trusted nodes aims to verify the correctness of locations reported by untrusted nodes.

Widely-deployed examples of positioning or verification schemes include radar systems, which are utilized by civil or military traffic management to find the positions of aircraft or ships. In turn, Global Navigation Satellite Systems (GNSS) are used for self-positioning by billions of devices and users around the world. These technologies also provide location awareness to a diverse set of applications such as traffic control, environmental monitoring and emergency services. Fueled by the global and free availability of GPS, self-positioning has become particularly popular in the transportation domain due to its excellent scalability and coverage. This development is accompanied with the need for location verification, especially in safety critical domains such as the aviation transportation [1,2].

One common approach to solving all three problems is to exchange signals between a node at an unknown or untrusted position and multiple nodes at known positions. Based on measurable physical signal effects such as propagation delay or path attenuation, the target node’s position is then estimated (or verified) by inserting the measurements into a model and by solving for the unknown location.

Generally, localization solutions discussed in the literature are evaluated either by using simulations or unique and difficult to replicate lab setups and scenarios. Consequently, it is hard to assess their performance in the real world and to maintain the core scientific principles of reproducibility and comparability. Live competitions have emerged as one potential solution to this problem for indoor localization, pitting a number of approaches against each other in a realistic setting [3,4]. However, in addition to the inherent difficulties in replicating such one-time events for non-participating approaches, they are very resource-intensive for organizers and participants, resulting in the exclusion of many potential solutions.

We argue that there are two main reasons for the lack of repeatable and realistic evaluations in outdoor localization research. First, real-world measurements can be expensive in domains such as aviation or shipping, as multiple high-quality receivers are needed which can cost up to USD 1000 or more. Second, researchers often lack access to a good variety of multiple sites over a large area as organizations with such infrastructures do not share their data.

Open and crowdsourced initiatives such as the OpenSky Network [5] (https://opensky-network.org/ (accessed on 10 July 2021)) exist to address these issues and have improved the availability of wireless data. However, their focus is generally on the data content. Access to and usage of physical layer information requires deep system and domain knowledge for the necessary complex pre-processing and data cleansing. Thus, their utility is currently limited for research into localization algorithms. In addition, the large size of these initiatives do not guarantee that a chosen sample is representative and comparable.

**Figure 1:**
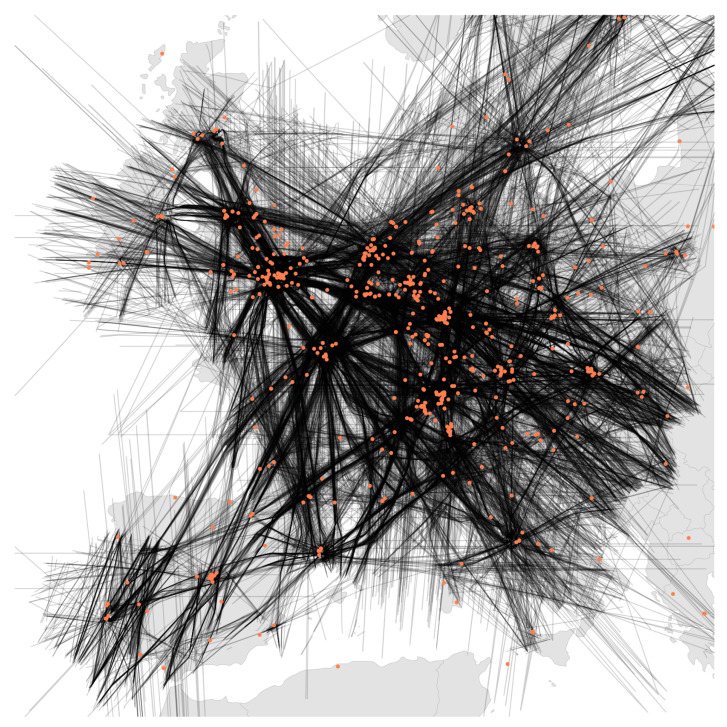
The LocaRDS data set with 50,865,291 aircraft positions (black lines) and 323 sensor positions (orange dots). In addition to geographic information, the data set contains time of arrival and signal strength measurements for each position reported by an aircraft.

Altogether, this results in complete fragmentation of localization research, where authors have to build their own test sets from real or simulated data in order to compare their own novel methods. In this paper, we solve this problem by presenting LocaRDS, a prepared and proven reference data set, which fulfills the scientific requirements of many localization methods. By using the established research network OpenSky, we create an open data set of real-world crowdsourced flight data that contain physical layer information of more than 50 million messages gathered from 323 sensors distributed over a large and diverse geographical area in Europe (see Figure 1 for a graphical illustration). The data set is suited for research in the areas of localization, location verification, positioning or other fields where time difference of arrival and signal strength measurements are needed.

Our larger mission with this work is to improve the state of the art in long-distance localization research and to place it on solid scientific grounding for future investigations. We hope that by using a comparable and standardized reference source, it becomes clear what the best solutions are to these problems under realistic conditions. Related to this goal, we discuss the requirements and the metrics that are requried to be implemented for such a data set in order to enable fair and useful comparisons.

The full LocaRDS data set is available for download at the Zenodo repository: https://doi.org/10.5281/zenodo.4298998 (accessed on 10 July 2021). It is published under the CC BY-SA license. The authors are members of the OpenSky Network association.

## 2. Application Areas

LocaRDS targets research where the following system and communication characteristics are common:

- **Location awareness:** Location information is needed and/or shared between network nodes. This often implies that (some) nodes are *mobile* since there is less of a need for constant location awareness in static networks;
- **Wireless communications:** Nodes use point-to-point radio links to establish and maintain location awareness, specifically in line-of-sight environments. Such wireless communications are typical, e.g., in mobile ad hoc networks;
- **Passive infrastructure:** Radio signals are received by a set of passive sensors. Moreover, a single signal should be received by multiple sensors;
- **Signal metadata:** Each receiver measures signal strength and/or time of arrival (ToA). These metadata can (but do not have to) be synchronized or otherwise calibrated.

LocaRDS is specifically targeted at (yet not limited to) three research domains where these characteristics are common: wireless positioning, self-positioning, and location verification. While all three methods can in principle be conducted with a wide range of physical measurements, such as the Doppler shift or angle of arrival, the most popular ones are time difference of arrival (TDoA) and received signal strength (RSS). We note at this point that the measurement of RSS is much more heterogeneous across different crowdsourced receivers and is provided `as is’ without any guarantees of quality. This is not the case with the TDoA, which we will focus on in the remainder of the paper.

### 2.1. Positioning

As wireless positioning is a widely applicable and popular research area, providing a complete overview here is beyond the scope of this work. As an introduction to the topic, Mao and Fidan [6] provide an extensive overview of the classical strategies and algorithms used in this area. The authors in [7] further systematize 29 ToA-based techniques, in particular, in non-line-of-sight environments, while [3] reports an indoor competition comparing 22 different teams. Other highly effective indoor-location methods exist, such as FM radio-based localization using the received signal strength [8] or the infrastructure-free fusion of geomagnetic and visual sensors [9]. However, of particular interest for us are positioning works such as [2,10], which consider passive outdoor line-of-sight environments.

### 2.2. Self-Positioning

A closely related but different problem is enabling a node to determine its own location; this is also known as self-positioning. Self-positioning approaches usually employ one or more moving beacon nodes that constantly broadcast signals. These signals are used by passive nodes to infer their own locations. Global navigation satellite systems such as GPS are a prominent real-world example of this system design.

Academic work on self-positioning (e.g., [11,12]) is mostly focused on indoor scenarios and this is likely due to the global availability of GNSS outdoors.

### 2.3. Secure Location Verification

In location verification, a set of nodes aims at verifying the correctness of location information provided by untrusted nodes [13]. Approaches follow a general pattern where location-dependent and tamper-proof physical signal propagation characteristics are measured and compared to a claimed location. Existing location verification methods can be broadly classified into angle of arrival, time of flight (or distance) bounding and time difference of arrival approaches.

While LocaRDS can support all three classes, e.g., by serving as input for simulations with realistic node movement and communication behavior, the class that can arguably benefit the most are TDoA-based approaches [2,14,15,16,17]. They rely on timestamps for the arrivals of a signal at different locations, which is something LocaRDS provides. Similarly, research in data validation (e.g., [18,19]) could benefit from LocaRDS, especially those with a focus on crowdsourced data.

### 2.4. Receiver Synchronization

Calibration and synchronization of the receiving sensors is also a subject of research as it is a key requirement across all three discussed research areas. Many of the solutions in these domains require very tight time synchronization, in particular, those based on TDoA measurements. This is costly even in controlled industrial deployments but it is impossible to achieve consistently with the variety of modern crowdsourced sensors used by enthusiasts to feed OpenSky and similar networks.

Consequently, any positioning method working in such an uncontrolled mixed-sensor environment must be able to process timestamp data of varying accuracy and provide good robustness against outliers. For example, the authors in [2] develop a solution based on the k Nearest Neighbor algorithm and show that it is less susceptible to noisy timestamps and imperfect sensor network geometry than traditional Multilateration (MLAT) approaches. Due to the dilution of precision effect in these algorithms, the issue of inaccurate timestamps becomes more problematic in crowdsourced deployments or, in general, where the relative positions of receivers and localization targets are non-optimized. For example, in [20], this problem was solved for MLAT implementation outside the airport by using a regularization method for the ill-posed problem.

## 3. Data Set Requirements

After summarizing the design aspects of positioning, self-positioning and location verification, we derive the requirements for the data set covering both the requirements with regards to the underlying system and the researcher requirements. We cover the overarching motivation of scientific comparability in detail in the following section.

### 3.1. System Requirements

For a consistent and useful data set, these requirements should be fulfilled by the underlying collection and processing system.

- **Real-world data:** Synthetically created data are not able to capture all complexities found in the variety of receiver setups and their respective environments. It is difficult to accurately model all factors influencing even a single controlled wireless receiver, let alone a host of heterogeneous and uncontrolled ones. Hence, only real-world data can provide the basis for our data set as it contains measurements which include all uncountable factors that influence communications and measurements in a realistic scenario.
- **Large-scale deployment:** In order to be able to conduct effective localization with sufficiently overlapping node coverage in various geographic and geometric scenarios, a large-scale deployment with hundreds of nodes with redundant coverage in most areas is crucial.
- **Variety in hardware and software:** In order to show the generality and wide applicability of the solution approaches, there should be different sensor types with different capabilities (e.g., time synchronization) providing the underlying data. Similarly, different software features and typical behavior should be reflected in the data set.

### 3.2. Researcher Requirements

We now consider the needs of research users and the widest accessibility of LocaRDS can be guaranteed, without which the goal of scientific comparability would fall short.

- **Ground truth available:** In order to judge the accuracy of an approach, there must be grounded truth about both the origin and the destination of a signal.
- **Documentation:** In order to understand the data set and to use it effectively in research, the underlying collection system must be described and all possible data and system artifacts should be known and documented.
- **Pre-processing:** The data needs to be preprocessed to remove unnecessary information and should be prepared in a form that renders it readily usable for a wide range of applications; the standard CSV format is the natural choice.
- **Variable scenarios:** Localization research comes in many different forms and uses a heterogeneous mixture of physical layer data and message content. These scenarios should be covered as well as possible, providing different parameters (e.g., states of synchronization, location information, mobile nodes, stationary nodes, different accuracy levels of ground truth, different geometries and different numbers of receivers).
- **Large number of measurements:** Modern machine learning solutions, in particular, those using deep learning (e.g., [21]) require a large number of raw measurements to use in training in order to be effective. This means providing millions of measurements to work with in any particular setting.
- **Quality indicators:** In order to make selection of subsets for specific scenarios easier, there needs to be an estimate about the quality of any particular measurement. This includes, for example, the quality of synchronization, clocks or timestamps.
- **Privacy:** In order to comply with ethical requirements, the privacy of all participating nodes must be respected.

### 3.3. Scientific Comparability

We follow recent efforts in the networking, transportation and security research communities (for example [1]) which aim at the better reproducibility of scientific experiments after the rise of what has been dubbed a “reproducibility crisis” in many scientific fields [22]. Existing positioning research has generally relied on individually-designed studies, either based on simulations or with data collected using the methods available to researchers and their collaborators. Typically, code and/or data set(s) are not available often due to intellectual property rights or other constraints imposed, for example, by industry collaborators in sensitive fields such as defense or aviation. While this approach means that solutions to positioning problems can be tailored specifically to their environments, it makes it impossible in practice to compare different approaches to one another.

Hence, the purpose of our data set is to provide a common baseline to compare large-scale localization solutions in the real world. This has several positive effects, from reducing the time and effort required for researchers to perform high-quality studies to better comparability for users interested in the advantages and disadvantages of the available solutions. While the use of LocaRDS cannot replace the in-depth analysis of a novel localization approach, we believe that the existing situation is a significant hindrance to the advancement of the field.

### 3.4. Related Work

Previously, the Evarilos project has attempted to address the issues of comparability and reproducibility in indoor localization by providing comparative evaluation scenarios in healthcare and underground mining [4]. However, the benchmark suite and data has become unavailable since, in order to prevent this fate, the LocaRDS data have been stored at Zenodo, which is a permanent repository of record.

There are few other works focused on creating and publishing dedicated datasets. The authors in [23] publish their used dataset in order to support reproducibility. They recorded audio signals from different locations to test their novel TDoA algorithm. In addition to the differences in application domains and signal origins, there is little documentation available on the collection and preparation of this size-restrained dataset. In the biological domain, there is the “Pipistrellus pipistrellus TDoA dataset” [24], a small dataset which contains audio recordings from bats. The same authors published another TDoA dataset, which is based exclusively on simulation [25]. The closest related dataset comes from a recent publication on ultra-wide band localization on small unmanned aerial vehicles (UAV) [26]. It contains measured TDoA, accelerometer and gyroscope data plus the ground truth of the UAV. As an indoor experiment with small flying objects, it is naturally limited in scope and size compared to LocaRDS. Beyond TDoA, Dvorecki et al. released the “Intel Open Wi-Fi RTT dataset” [27], which includes almost 30,000 Wi-Fi RTT raw channel measurements from real-life client and access points. Based on data collected in an office environment, it can be used for indoor localization research.

To the best of our knowledge, LocaRDS is thus the first localization dataset that is built by the use of large-scale, long-range and real-world signal data and specifically aimed at scientific reproducibility and comparability.

## 4. LocaRDS: The Localization Reference Data Set

In order to meet the requirement of documentation, we describe how LocaRDS was collected and prepared. This knowledge is key to understanding which processing and system artifacts are to be expected in the data. We will then provide an overview of the structure and contents of LocaRDS.

### 4.1. Collection

The raw data used to generate LocaRDS was recorded by the OpenSky Network (OSN) [5]. The OSN is a network of more than 2500 crowdsourced sensors, which collects air traffic control data at a large scale and it provides these data to researchers for free. The network records the payloads of all 1090 MHz secondary surveillance radar downlink transmissions of aircraft along with the timestamps and signal strength indicators provided by each sensor upon signal reception.

Part of this data set are the exact aircraft locations that are broadcasted twice per second by transponders using the Automatic Dependent Surveillance-Broadcast (ADS-B) technology. With respect to the requirements discussed in Section 3, we are only interested in this position reporting a subset of ADS-B, as it provides the GNSS-derived locations of the transmitters which serve as the necessary ground truth. In addition to this measurement data, the OSN provides a list of all sensors, including their locations and device types.

Together, these two data sets (measurements and sensor information) constitute a well-suited basis for LocaRDS. In particular, we highlight several properties relevant to localization, self-positioning and location verification problems:

- **Known locations:** The locations of all nodes (transmitters and receivers) are known. This provides the ground truth and reference locations needed by many algorithms.
- **Coverage redundancy:** Each transmission is received by multiple receivers. Since this is a key requirement for most localization algorithms, we limit LocaRDS to data recorded in Central Europe where OpenSky’s redundancy is highest and its coverage is nearly complete.
- **Diversity:** There are thousands of different transmitters constantly broadcasting their locations, while hundreds of different receivers are recording these signals. This provides a rich set of measurements with varying accuracy and geometry, which in turn allows researchers to test the influence of different factors on the performance of their algorithms.
- **Mobility:** The transmitting nodes (i.e., the aircraft) are moving through the network at variable and typically very high speeds of 800 km/h and above, which creates a highly dynamic network topology.
- **Crowdsourced:** The data comes from a crowdsourced system of receivers, integrating all the challenges and difficulties found in such an organically grown and non-controlled set of receivers. Collecting data from a synchronized and controlled deployment would be less flexible and less widely applicable. Conversely, due to the high sensor density and high level of redundancy in the OSN, the selected subsets of this data can emulate controlled deployment.

### 4.2. Preparation

In order to make the data accessible and to meet the requirements, complex pre-processing is needed. It is necessary to create a data set independent from the system aspects, to reduce the data volume and to reduce, as much as possible, the need for the researchers to know all original system details. Moreover, the information quality needs to be assessed and indicated, allowing researchers to choose subsets of data that match their own requirements. Therefore, we performed the following processing steps to prepare the unstructured OSN data and created a well-defined data set for scientific comparisons:

#### 4.2.1. Decoding

Decoding ADS-B correctly is a complex task. Although libraries and tutorials such as [28] exist, it remains a tedious task that requires a deep understanding of the underlying link layer technology, Mode S. Moreover, the sheer volume of data collected by OpenSky (about 120 GB of raw data per hour) makes this process challenging and resource-intensive. Therefore, we relieve researchers from this burden by providing readily decoded location information in WGS84 coordinates, altitude information in meters and a unique aircraft identifier as a simple integer.

#### 4.2.2. Continuous Timestamps

Timestamps are provided in different resolutions and units depending on the receiving sensor type. Moreover, some sensors only provide rolling counters, counting from zero to some power of two at a certain frequency. In order to abstract this, we implemented a counter overflow detection mechanism and mapped all timestamps to a continuous timestamp with a common unit (nanoseconds). The complexity of this preparation step comes from the fact that raw data coming from a sensor can have an unknown and varying delay, making estimation of overflows difficult.

#### 4.2.3. De-Duplication

OpenSky’s raw data are merely long lists of single measurements by single sensors. However, as most localization algorithms rely on signals being received by multiple receivers, we grouped multiple receptions belonging to the same transmission event. This process is called deduplication. We note here that this is performed for both data from GPS-synchronized and non-synchronized sensors since we are not using timestamps. We are exploiting the fact that aircraft are constantly moving and their three-dimensional positions are fairly unique. Hence, we can group signals simply based on the payload of the ADS-B signals. However, a small number of falsely grouped measurements remains as noise in the data since avionics sometimes do not update their location fast enough between two transmissions, which results in transmitting the same location more than once.

#### 4.2.4. Filtering

To render the data set more manageable without losing temporal or topological properties, we reduced the input data set to ADS-B position reports received in Europe, where OpenSky’s coverage and redundancy is by far the best. Moreover, we discarded transmissions received by only one sensor since they have little value for the purposes of LocaRDS and omitting them reduces the size of the data set significantly.

#### 4.2.5. Quality Assessment

Crowdsourcing creates several issues regarding the quality and integrity of location and timing information of certain aircraft and sensors [19]. In order to allow researchers to ignore these effects while still preserving them as a potential subject of research, we implemented integrity checks from [19] to verify and judge the data correctness and added respective indicators to LocaRDS. In principle, our data validation first detects GPS-synchronized sensors with accurate location information based on their timestamps. These GPS-synchronized timestamps are then used to verify the location information provided by different aircraft.

### 4.3. Available Information

The following information is available in LocaRDS:

- **Transmitter location:** The location of the aircraft at the time of transmission. Locations are provided as WGS84 coordinates in decimal degrees. There are two altitude values provided, which are barometric and geometric (WGS84). While geometric altitude is needed in most cases, often only barometric altitude is transmitted in the ADS-B messages in real-world air traffic control scenarios.
- **Receiver location:** WGS84 coordinates of the receiving sensor. Note that locations can be inaccurate for different reasons, sometimes with high offsets to their real location [19]. However, it can be assumed that the vast majority of locations are accurate. In addition, to handle the cases with high offset, we provided indicators marking verified locations, which we discuss further below.
- **Sensor timestamp:** Timestamp measured by the sensor at the time of signal arrival in nanoseconds since the beginning of the recording. Depending on the sensor type and the setup, timestamps highly vary in quality and they might be subject to drifts of varying degrees or even be broken (e.g. constant or random values). The accuracy of timestamps depends on many often unknown factors such as sample frequency, signal-to-noise-ratio or software configuration. Refer to [19] for more information.
- **Received signal strength indicator (RSSI):** Signal strength as measured by the receiver in dB with unknown reference. RSSI calibration depends on unknown factors such as antenna gain, device type, gain settings, etc. Since calibrating the RSSI is beyond the scope of this work, users who require calibrated values need to devise and apply a calibration method (e.g., based on the well-defined transmission power of ADS-B and the free-space path loss model).
- **Server timestamp:** Timestamp measured by the server when the ADS-B position report was first observed in microseconds since the beginning of the recording. This timestamp can be used, for instance, in time synchronization methods. Note that it includes the unknown and highly varying internet delay between the sensor and OpenSky’s server.
- **Aircraft location accuracy indicator:** Binary per-aircraft indicator for the quality of the provided locations. The accuracy ADS-B positions depends on the availability of GPS on-board the aircraft. As ADS-B is still in its final deployment phase, some aircraft report locations with an offset or with a delay. This Boolean quality indicator helps users select reliable transmitter locations, e.g., as ground truth. If this indicator is false, then the location provided by the aircraft is known to have low quality. A missing indicator means that the aircraft could not be verified due to bad geometry or a lack of reliable measurements.
- **Sensor location accuracy and synchronization indicator:** Binary per-sensor indicator for the time synchronization status and accuracy of the location information of a sensor. As timestamps and sensor locations are jointly used to assess their quality, errors cannot be attributed to either of them reliably. Hence, this flag is only true if the receiver-provided timestamps did not drift over the course of one hour and the sensor location could be verified based on these timestamps.

### 4.4. File Structure and Format

**Figure 2:**
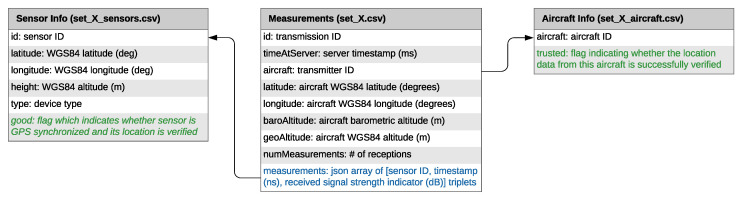
Structure of each 1 h subset of LocaRDS. Each subset consists of three files. The main file set_X.csv (X = 1...8) contains all measurement data. Each measurement is associated with multiple entries in the sensor information file (set_X_sensors.csv) and exactly one aircraft data validation result (set_X_aircraft.csv).

LocaRDS is split into eight subsets, each containing one hour of data (different weekdays/time of day). The duration was chosen as a trade-off between data volume per subset and possessed continuous flight movements covering longer distances of up to about 800 km. As a general file format, comma-separated values (CSV) were chosen for its wide support among data science tools and programming languages. Each subset with identifier X consists of three CSV files. The file **set_X.csv** contains all ADS-B position reports that were received by at least two sensors, including the measurement data (timestamp and signal strength indicator) for each transmission. Location, GPS synchronization status and location accuracy indicators of all sensors can be found in **set_X_sensors.csv**. Finally, **set_X_aircraft.csv** contains the location verification results indicating whether the location information provided by this aircraft is of high accuracy or not. An overview of structure, formats, columns in each CSV file and links between the files is provided in Figure 2.

## 5. LocaRDS Data Set Characteristics

In total, LocaRDS contains 222,501,602 single measurements from 323 sensors split into eight 1 hour chunks. The total size of the CSV files is 9.31 GiB. As mentioned in Section 4.2, all time and signal strength measurements referring to the same transmission are grouped during the de-duplication process, resulting in 50,865,291 groups. In addition, whenever possible, the locations reported via ADS-B are verified based on the timestamps provided by GPS-synchronized sensors, as explained in the following subsections. Overall, there are 11,894,627 transmissions from verified aircraft positions. An overview over the size and distribution of the data across the chunks is provided in Table 1.

### 5.1. Geographic Distribution

All aircraft and sensor locations in LocaRDS are shown in Figure 1. The major European hubs such as London Heathrow, Frankfurt International and Paris Charles De Gaulle are clearly visible since most trajectories converge towards them. The sensor density in LocaRDS is higher around major airports. This is highly beneficial for localization research since, due to the line-of-sight limitation of 1090 MHz communications and the Earth’s curvature, ranges at lower altitudes are shorter. Hence, higher sensor density is required close to airports where arriving and departing traffic is moving at low altitudes. Since these trajectories are more dynamic than en route traffic (which is mostly flying straight lines), low-altitude traffic might be of special interest to some researchers.

### 5.2. Measurement Redundancy

**Figure 3:**
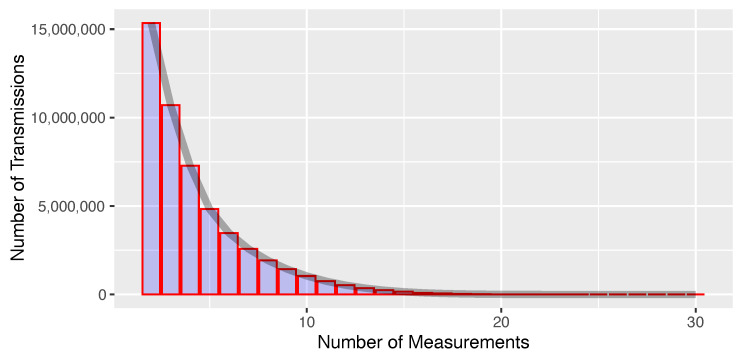
Distribution of number of measurements per transmission by following a geometric distribution with a success probability of 28%.

**Figure 4:**
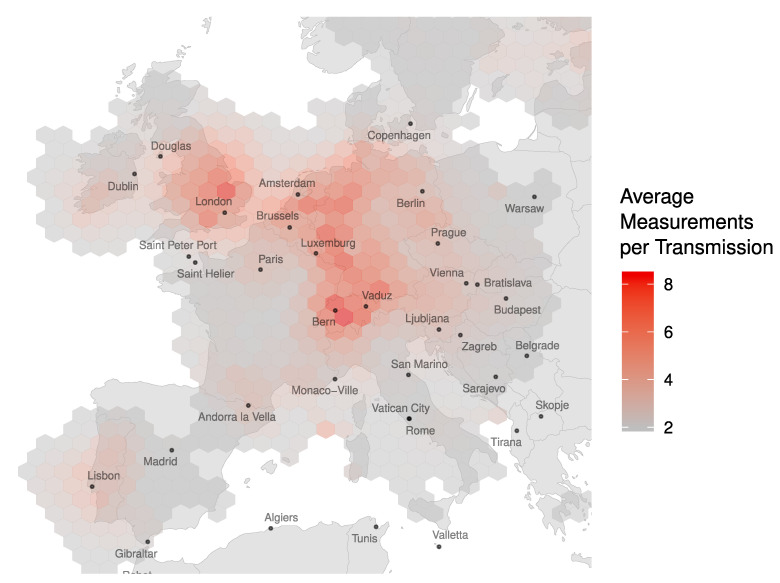
Distribution of the average number of measurements per LocaRDS transmission. The number of receivers for a single transmission ranges from 2 to 30 depending on OpenSky’s coverage redundancy. It is the highest in Central Europe, with an average of 8.

Each ADS-B position report included in LocaRDS was received by at least two sensors. As shown in Figure 3, the number of measurements per transmission follows a geometric distribution with a maximum of 30 sensors receiving the same transmission. The geographic distribution of coverage redundancy is shown in Figure 4.

### 5.3. Data Verification

While we do not have direct access to the measurements of the GNSS receivers onboard the aircraft, which would be required in order to have fully reliable ground truth data, we can rely on the fact that, statistically, the quality of the data is sufficient for air traffic control to conduct their work in a safe and reliable manner. Additionally, we use a verification procedure in order to ensure the quality for a particular subset of the data as far as possible in a crowdsourced system.

Verifying the accuracy of the data without having a reliable ground truth—neither for aircraft nor for sensors—poses a chicken-and-egg problem. In order to verify aircraft information, we need reliable sensor information and vice versa. However, we can make the assumption that the majority of position information coming from ADS-B equipped aircraft is fairly accurate. As mentioned in the previous paragraph, this assumption is generally reasonable given the high integrity, accuracy and system design assurance levels that are used in aviation. Moreover, we know that we have a small yet significant number of GPS synchronized sensors in our network. We use these two assumptions to verify both aircraft and sensor information by applying the following steps.

First, we identify sensor pairs for which their clocks are synchronized by calculating a linear regression for the timestamps of the two sensors. If the resulting slope is 1±10 ns and the root-mean-squared error (RMSE) is below 1000 nanoseconds, we assume that the two sensors are synchronized with each other. We generalize this pair-wise information to single sensors by testing whether a sensor is synchronized with at least two other sensors. If that is the case, we flag the sensor as GPS-synchronized. In order to also include GPS-synchronized sensors that only have overlapping coverage with just one other sensor, we calculate the transitive closure of this initial result by also flagging sensors as GPS-synchronized if there was at least one pair of that sensor with a GPS-synchronized sensor with a slope of 1±1 ns and an RMSE smaller than 1000 ns.

In the next step, we use the GPS-synchronized sensors to verify the aircraft positions. We first calculate for all TDoA measurements of GPS-synchronized sensors the TDoA values that would be expected if both receiver and aircraft locations are correct. Then, we select those aircraft where the root-mean-squared expected TDoA value across all sensors is at least 200 µs. This filtering step makes sure that no aircraft are verified that were only tracked by sensors which are very close to each other. Sensors that are very close cannot provide any meaningful information regarding the accuracy of an aircraft’s reported positions since they will always have a TDoA close to zero regardless of the aircraft’s position. Finally, if the absolute median of deviation between expected and measured TDoA was below 100 ns and the standard deviation was below 200 ns, we mark those aircraft as trusted.

**Figure 5:**
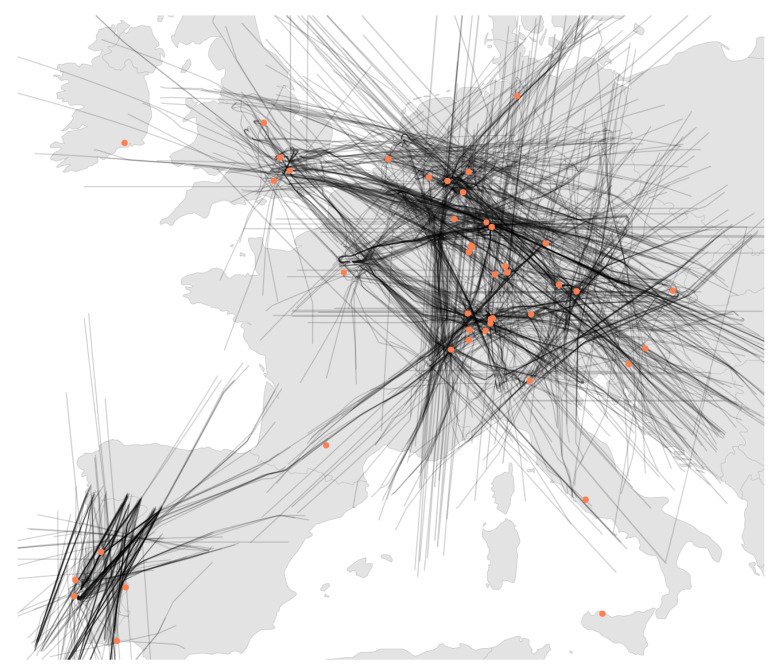
Verified aircraft and sensor positions in LocaRDS. The subset was created by using the provided indicator that measured the reliability of location and timing information from a specific aircraft and sensor.

About 12% of the aircraft locations and 22% of all measurements in LocaRDS are verified. However, this does not mean that 88% of the reported positions are incorrect. In fact, a large fraction of the data (61%) could not be verified due to bad geometric conditions (bad dilution of precision) or the lack of synchronized sensors. Since the above data verification algorithm applied to LocaRDS relies on coverage redundancy and cross-checks of data coming from different receivers, the geographic distribution of the LocaRDS subset of verified aircraft positions and sensors is also concentrated around the hot spots in Figure 4. The set of all verified aircraft and sensor positions is shown in Figure 5.

#### Receiver Performance

In OpenSky, about 80% of all feeders use cheap RTL-SDR and Raspberry Pi setups, which do not support GPS synchronization and cost broadly in the range of $50–100. Conversely, fewer than 20% use setups which are about one order of magnitude more expensive and offer relatively tight synchronization via GPS. Moreover, the accuracy of these setups is dependent not only on the availability and quality of time synchronization but also by the resolution of the internal clocks and the sampling process. The latter can vary significantly from 2–2.4 MHz for an RTL-SDR dongle vs. about 60 MHz for a more expensive Radarcape) and, thus, influences the level of noise that time-based localization solutions have to deal with. Note that, although related, timestamp resolution is not equal to accuracy.

### 5.4. Data Anonymization

All contributors to OpenSky have the option to decide whether their accurate sensor location data may be distributed for research use or not. For those opting to participate, we have anonymized their sensor IDs for LocaRDS. Likewise, we have anonymized all unique transponder IDs of transmitting aircraft, although the distribution of historical track data are not generally a concern for most, as their wide availability demonstrates. It has to be noted that some of this information could be recovered if an adversary had access to the underlying OpenSky data or other large-scale databases and additional knowledge, for example, of the time frame of the data collection, but we consider the practical risks of this issue as negligible.

## 6. LocaRDS Example Localization Implementation

As it is difficult to comprehensively provide tight time synchronization in crowdsourced networks with low-cost receivers, we propose here an initial (TDoA-based) synchronization and localization procedure for dealing with the data. It is composed of two parts: an opportunistic synchronization algorithm and a hyperbolic localization algorithm. The synchronization algorithm exploits ADS-B position messages to continuously estimate the time offset and drift between any two sensors that share air traffic communication and the hyperbolic localization algorithm exploits these paired sensors to continually estimate the positions of aircraft emitting any type of signal with measurable ToAs.

This combined approach is capable of locating ADS-B transmitters or, in general, aircraft emitting any kind of radio signal for which is possible to measure the time of arrival. Moreover, it does not need an external synchronization method (e.g., GNSS, reference transponder, NTP, or others) and is robust to outliers and anomalies (such as temporary offline sensors or measurement integrity issues).

### 6.1. Sensor Pairing and Offset estimation

It is hard to synchronize all sensors in a controlled wide-area or global sensor network to a sub-microsecond level; it becomes practically impossible in the case of crowdsourced networks with no control over heterogeneous hardware and sensor setups. Additionally, each sensor can be switched on or off at any moment.

For these reasons, we exploit a continuous synchronization method to estimate and track the clock offset and drift for each possible pair of stations that share the same traffic. In particular, for each pair of sensors receiving the same position message from an aircraft, the clock offset is measured by measuring the ToAs of the message at each sensor ($t_{i}$,$t_{j}$) and knowing the aircraft position p (e.g., from ADS-B position message decoding) and the positions of the sensors ($s_{i}$,$s_{j}$):

(1) $$\Delta t_{i,j}=\left(\frac{∥s_{i}-p∥}{c}-\frac{∥s_{j}-p∥}{c}\right)-\left(t_{i}-t_{j}\right)$$

By repeating this measurement for all messages from aircraft in common view, a time series $\left\{\Delta t_{i,j}\right\}$ for the paired sensors’ offset is obtained and tracked in time to estimate current offset and drift and to forecast their values for any future time. More details about ADS-B sensor clock tracking via ADS-B messages can be found in [29], where a set of Kalman Filters are used to track the clock biases and drifts of different ADS-B stations. However, any other tracking algorithms [30] can be used. (In case of offline or a posteriori localization, the real-time Kalman Filter estimator/predictor can be replaced with any interpolation, fitting and smoothing algorithm.)

If more than two stations are in common view of the same aircraft, the offsets and drifts for any possible pairs are estimated for the entire time that they both receive the aircraft messages.

**Figure 6:**
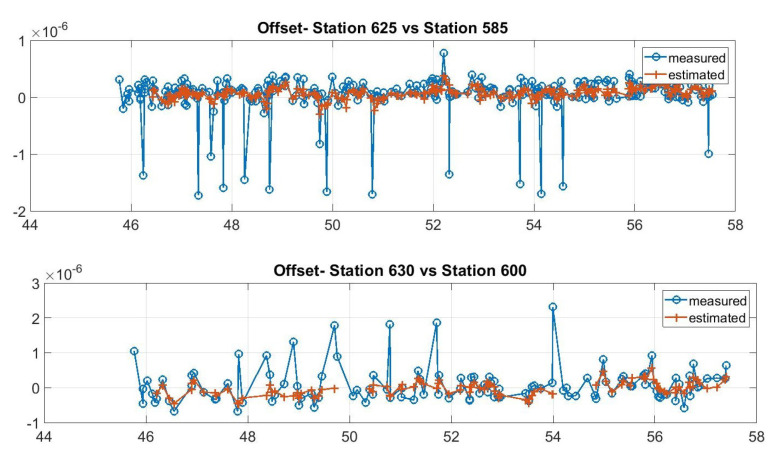
Examples of tracked offsets across two stations. Top (stations 625 and 585): measurements with some outliers (corrected by the tracking filter). Bottom (stations 630 and 600): measurement with outliers (filter re-initialization with some estimation gaps).

It is noted that with this method, the network does not have full synchronization, i.e., there is no single common reference time. Moreover, sensors that do not share any ADS-B traffic cannot be synchronized. This is not a problem for our application, as these are also not helpful in positioning aircraft. On the other hand, this method is simple and robust as it requires neither a complex time transfer mechanism, a common shared reference time system nor physical reference stations. In crowdsourced sensor networks, this provides several concrete advantages. First of all, if a station has a service interruption and transmits no data or incorrect data, this will not affect the other pairs’ synchronization. Second, even in cases where an aircraft transmits incorrect data, it will affect only the sensors within its range. Moreover, if these stations also have other aircraft in their common range, these incorrect data will be discarded as outliers through a subsequent pass of the tracker using innovation tests defined in [29,31]. In particular, when the innovation (i.e., the difference between expected and incoming measurements) is larger than a given threshold, the incoming measurement is not used and the offset is extrapolated by the previous state. If the threshold is exceeded several consecutive times, the tracking algorithm is re-initialized, i.e., the track is dropped and a new one is created exploiting the incoming measurements that do not match with the previous track. Examples of the offset tracking performance (in the case of measurements outliers and errors) are shown in Figure 6.

Finally, the proposed pairing procedure can be applied to any kind of receiver (GPS synchronized or not), obtaining absolute GPS time synchronization of all non-GPS station with at least one aircraft in common view with a GPS-equipped station.

### 6.2. Hyperbolic Localization Using Paired Sensors

Classical MLAT algorithms assume all sensor clocks are synchronized to the same reference station, i.e., full network synchronization. Under this assumption, four ToA measurements (i.e., three synchronized TDoA measurements) are sufficient for 3D localization of the target. As discussed before, this assumption (full network synchronization) does not hold in a crowdsourced network and our proposed approach is able to produce only synchronized sensor pairs. For this case, a modified hyperbolic localization algorithm is proposed here; for any aircraft to be localized, all possible pairs of previously synchronized sensors in view are identified, rearranging Equation (1) as follows.

(2) $$TDoA_{i,j}=\left(t_{i}-t_{j}\right)=\left(\frac{∥s_{i}-p∥}{c}-\frac{∥s_{j}-p∥}{c}\right)-\Delta t_{i,j}\;$$

Now $\Delta t_{i,j}$ is predicted by the tracking filter mentioned in the previous subsection and p is the unknown to be estimated. If more than three pairs of sensors are in the range of the aircraft, the corresponding equations can be used to find the airplane position. In this manner, the algorithms exploit independent pairs of sensors and do not need the network to fully synchronize.

**Figure 7:**
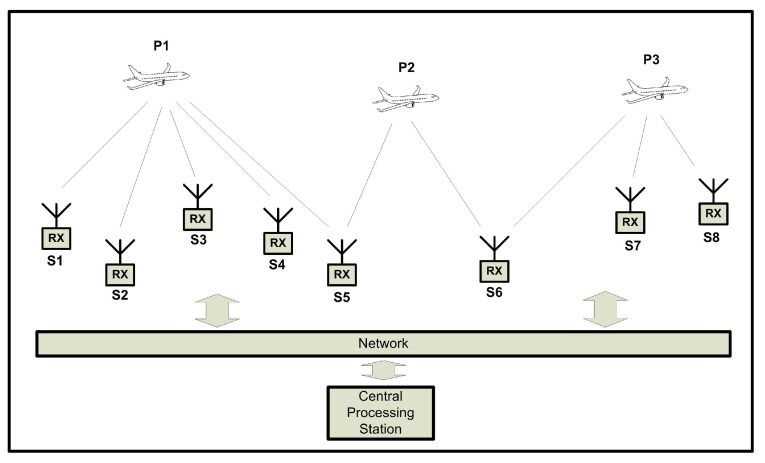
Illustration of reference implementation with sensor pairing.

A overview of this process is shown in Figure 7. The aircraft in position P3 can be used to synchronize the pairs $\left\{S6,S7\right\}$, $\left\{S6,S8\right\}$ and $\left\{S7,S8\right\}$ and the aircraft in P2 can synchronize the pair $\left\{S5,S6\right\}$. The four pairs $\left\{S6,S7\right\},\left\{S6,S8\right\},\left\{S7,S8\right\},\left\{S5,S6\right\}$ can then be used to localize any airplane communication that is received by stations (S5,S6,S7,S8), producing four different equations.

While some equations could be linearly dependent (for example, $\left\{S7,S8\right\}$ is a linear combination of $\left\{S6,S7\right\}$, $\left\{S6,S8\right\}$), if at least three equations are independent of one another, the aircraft position can be estimated.

In order to solve this system of non-linear equations, any classical method can be used. For example, if an approximate starting estimate of the aircraft’s position is known, the Newton–Raphson method can be applied.

The method for the initial estimate could be as follows:

1. If no estimation of the aircraft position is present from previous observations, the average of the sensor positions in range of the aircraft is used. This average can be weighted by signal strength (cf. the Centroid algorithm [32]);
2. Otherwise, the last estimate of the aircraft position is used.

**Figure 8:**
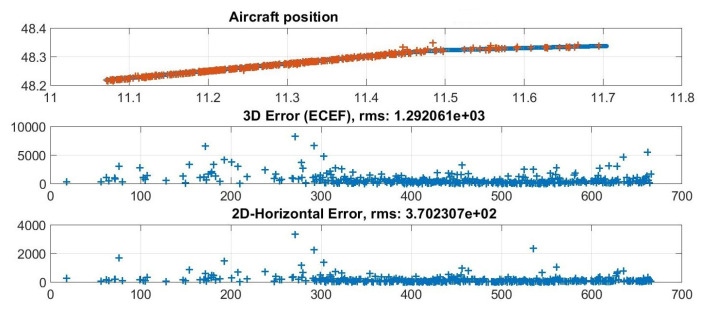
Basic illustration of the localization performance. From the top: latitude and longitude of a single tracked aircraft (blue: ADS-B; red: estimated positions); 3D error in meters; 2D error in meters.

An example of the algorithm performance is reported in Figure 8 which shows an aircraft track and its horizontal and three-dimensional errors (w.r.t. the ADS-B data).

## 7. Discussion: Evaluation and Metrics

We now discuss some metrics that can be used to evaluate algorithms that use LocaRDS. There is no claim to completeness; on the contrary, we expect this be an ongoing process, which we will actively support through the OpenSky website and foundation. The metrics chosen for the scientific evaluation of localization should be as broadly applicable to different scenarios and methodologies as possible.

### 7.1. Localization Accuracy

The key metric for localization is the accuracy with which the position of the target is predicted. While the utility of aircraft localization depends very much on the context and the use case, (There are 12 categories of navigational accuracy defined in the ADS-B protocol, ranging from <3 m to >10 nautical miles.) higher accuracy is strictly better. RMSE has been widely included as a standard metric to compare the predictive performance of localization models (see, e.g., [3]).

Unfortunately, RMSE can be affected by a small number of outliers with large location errors. This can often happen in crowdsourced sensor networks, in which, among other things, the geometrical position of the sensors is not optimized. We propose to mitigate this effect by use of the Truncated Root Mean Square Error (TRMSE) . The TRMSE is obtained as follows: the square error for each position is computed and then the largest errors are excluded. The percentage of the measurements to be excluded or the error threshold to be used for the exclusion can be dependent on the research being conducted.

### 7.2. Data Set Coverage

The second consideration concerns the coverage of the evaluation data sets, i.e., how many of the data points of LocaRDS were chosen to be predicted. While ideally all samples would have a prediction, this is not practical for several reasons. For example, some methods may need initial samples to calibrate and also regularly re-calibrate. Furthermore, there is also value in correctly choosing to not predict bad or uncertain samples in order to minimize outliers and improve the average localization performance. However, it is obvious that with equal localization accuracy, higher coverage is strictly better.

Concretely, a threshold on the minimum number of aircraft position predictions should be defined considering the application requirements and also considering the sensor coverage in a given geographical region.

### 7.3. Further Considerations

Due to the variation in the distribution of uncertainty and quality of measurements in LocaRDS, it is clear that there can be trade-offs between high coverage and high accuracy. In addition to requiring a certain minimum coverage, this trade off can also be quantified for a provided solution by applying a penalty directly towards the accuracy scoring. By assuming a fixed high localization error for any missing observation, the overall RMSE (or TRMSE) is increased. However, the effectiveness of the penalty is highly dependent of the quality of the provided solution: If the penalty is set below the RMSE, it will actually improve the quality score and thus set a false incentive to leave out observations. Hence, this penalty should be adjusted by keeping in mind the average quality of the solutions.

A second consideration is centered around the run times of possible solutions. While the speed of localization algorithms is not crucial in many application scenarios and most modern computing architectures should be able to fulfill any real-time constraints, it may still be insightful to analyze. For example, variations in training times for machine learning based solutions may impact the choice of algorithms in situations where regular re-training is required. Similarly, lightweight algorithms for distributed resource-constrained edge computing are a relevant application, for example, for crowdsourced flight tracking networks.

## 8. Conclusions

In this paper we introduced LocaRDS, which is a reference data set for localization research. We derived the requirements for such a reference data set and showed how LocaRDS can successfully be used to test, analyze and directly compare localization techniques. While in the present work, we applied LocaRDS to the open research problem of aircraft localization in crowdsourced networks, we postulate that its appeal is much broader. For future work, we hope that many researchers consider LocaRDS for their own problems in the fields of positioning, self-positioning and location verification. We believe that results obtained with LocaRDS have a broad scientific and practical application and can offer a better methods going forward.

## Figures and Tables

**Table 1:** Information about the LocaRDS data set. GPS/ADS-B sensor numbers are not additive as they contribute to several subsets.

Set	Data Points	De-Duplicated	Verified	Sensors	GPS	GiB
1	28,234,130	6,457,542	1,839,760	318	45	1.18
2	28,717,685	6,535,444	1,680,956	317	45	1.20
3	28,749,671	6,569,830	1,996,987	318	44	1.20
4	28,215,712	6,348,679	1,810,382	317	43	1.17
5	26,313,445	6,111,569	1,452,447	314	41	1.11
6	27,360,671	6,309,260	540,953	313	40	1.15
7	27,514,781	6,345,589	779,524	313	39	1.16
8	27,395,507	6,187,378	1,793,618	309	42	1.14
All	222,501,602	50,865,291	11,894,627	323	46	9.31

## Data Availability

The LocaRDS data set has been made permanently available on the Zenodo repository: https://doi.org/10.5281/zenodo.4298998 (accessed on 10 July 2021). It is published under the CC BY-SA license.
